# From surfactant to cellulose and DNA self-assembly. A 50-year journey

**DOI:** 10.1007/s00396-016-3927-2

**Published:** 2016-08-22

**Authors:** Björn Lindman

**Affiliations:** Physical Chemistry, University of Lund, P.O. Box 124, 221 00 Lund, Sweden

**Keywords:** Surfactants, Polymers, Aggregation, NMR, Phase behaviour, Cellulose

## Abstract

Surfactants have been the basis for applications in several industrial sectors for a long time. However, fundamental research was 50 years ago still limited to a small number of academic groups and even basic aspects were controversial. The field has since undergone an enormous expansion and the improved understanding has laid the basis of numerous new products as well as been the basis of important parts of nano-science and -technology.The present author has during 50 years in academia devoted most of his research to amphiphilic compounds, including both surfactants and polymers. Hereby, I had the privilege of following a very exciting development. In 2015, I had the honour to receive the Life-time Achievement Award of IACIS, the International Association of Colloid and Interface Scientists. IACIS organizes since the 1970s a tri-annual symposium, typically the best attended in the field. For the first time since 2000, it was in 2015 organized in Europe, namely Mainz, Germany. This treatise is based on my award lecture in Mainz, which covered developments from my first research as a new Ph D student in Stockholm to current work as an emeritus and visiting professor. Interestingly, discoveries in my very early work contributed to solving problems in now on-going research. Håkan Wennerström kindly wrote a quite comprehensive paper about my achievements a few years ago (Adv Colloid Interf Sci 205:1–8, [1]). In writing the present paper, I have strived at covering mainly topics not treated in detail by Håkan. In fact, I will emphasize very much our early studies as well as our studies of surfactant self-assembly by NMR and in particular look at the developments of our research and connections between different research topics.

## Cellulose dissolution and regeneration

**Fig. 1:**
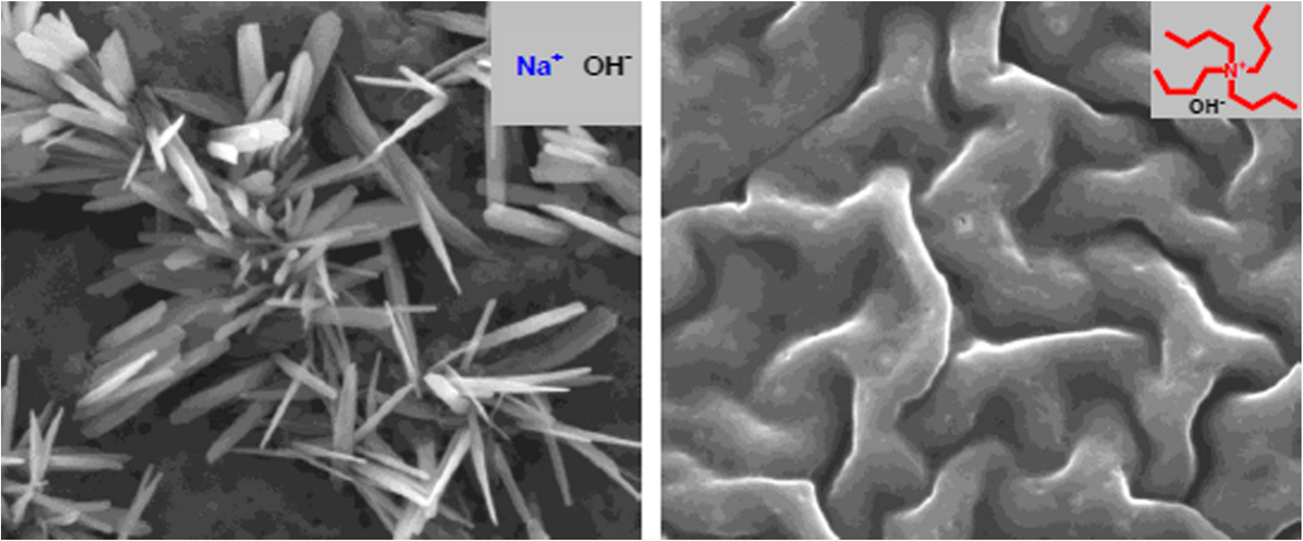
The morphology of regenerated cellulose depends strongly on the solvent used, here illustrated for two alkali solvents one with inorganic counterion and one with organic. Analogous differences are seen in X-ray diffraction. Reprinted from reference [6] with permission from Elsevier. For details see references [6, 7]

A few years ago, I was approached by an old friend and entrepreneur who wanted to start a new industrial project, where dissolution of cellulose from wood was crucial. For his purpose, current commercial processes (viscose and using N-methyl-N-morpholine oxide, NMMO) were not satisfactory from environmental and safety points of view. Instead, he was searching for a water-based solvent and asked me for agents that could break hydrogen bonds between cellulose molecules. Thus the common learning was that cellulose is insoluble in water because of strong intermolecular hydrogen bonding and that solvents like ionic liquids and NMMO act by breaking hydrogen bonds. As water has particularly strong hydrogen bonds and cellulose–water hydrogen bonds are similar in strength to those between cellulose molecules, I argued that to look for “hydrogen bond breakers” was probably not a suitable approach. Instead, other interactions must be involved and I suggested that hydrophobic interactions are relevant and that cellulose is amphiphilic. At his request, I went through the literature and could easily confirm that additives that weaken hydrophobic interactions or are amphiphilic can strongly influence the solubility of cellulose. Cellulose dissolution certainly involves the breaking of hydrogen bonds but it was sometimes overlooked that on dissolution new interactions of similar strength are formed. My short report for industry was reused after slight modification when I was approached to contribute to a Festschrift for a colleague at Coimbra University [2]. The interest this short paper received both from industry and academia suggested new research. The offer of industrial support as well as the fact that one of our many joint Coimbra–Lund Ph D students, Bruno Medronho, was just finishing and looked for a new challenge made for a very easy start of new research. Under Bruno’s direction, a research program focused on cellulose dissolution and regeneration as well solution structure has been established. In this work, several new water-based solvent systems have been developed and the amphiphilicity of cellulose confirmed, both in new studies by Bruno and students and by reinterpretation of work in literature. For example, there were reports that urea facilitates cellulose dissolution (see for example Cai et al. [3]), an observation attributed to a particularly efficient breaking of cellulose–cellulose hydrogen bonds. However, since urea is well-known protein denaturant and also inhibitor of surfactant self-assembly, we argued that more likely urea exerts its effect on hydrophobic interactions between cellulose molecules. Bruno could also show that surfactants strongly affect cellulose–cellulose association. The controversial aspects of this work are illustrated by two papers in Cellulose [4, 5]. In addition to that of the importance of hydrophobic interactions, two other problems have been in focus. One is the significance of ionization of cellulose in alkaline and acidic solvents, the other is the state of cellulose in solution. Bruno’s work with Daniel Topgaard and Luís Alves shows that it can depend strongly on the solvent used [6, 7] (Fig. 1). The exciting work on cellulose interactions and applications, composites, dispersions, etc., is continued under the direction of Bruno in Portugal and Magnus Norgren and Håkan Edlund in Sundsvall, Sweden.

Basic aspects and applications of cellulose have received a renewed interest worldwide in view of sustainability aspects. In starting research on cellulose, we were very much motivated by on-going activities around the globe. Here should be mentioned contacts with Patrick Navard, Thomas Heinze, Karin Stana-Kleinschek, Volker Ribbitsch and others at the European Polysaccharide Network of Excellence (EPNOE) [8–10] and with Lars Wågberg and colleagues at the Wallenberg Wood Science Center [11]. Important developments in this area have been taking place in the group of Lina Zhang at Wuhan University [12, 13]. Here, should also be mentioned Japanese contributions [14, 15].

Interestingly, in working on this my latest project, I found two direct connections to my very first research as a new Ph D student in Stockholm exactly 50 years ago:

1. Tetrabutylammonium hydroxide (TBAH) solutions are very good solvents for cellulose (cf. Fig. 1). In general acids and bases with organic counterions are good solvents.
2. One of the arguments advanced for the “hydrogen-bond breaking” mechanism of cellulose dissolution came from Cl NMR [16, 17]. Thus very large chloride ion NMR relaxation effects were attributed to interaction with the –OH groups in cellulose. However, in our early work, we had found the opposite: strong relaxation by nonpolar groups but not by polar groups.

## Electrolyte solutions: large ions bind to hydrophobic sites. Hofmeister series

After growing up in Uppsala and going to school there, I made my compulsory military service. My university studies started in 1962 in chemical engineering at the Royal Institute of Technology in Stockholm. At an early stage, I was attracted by the research atmosphere in the departments of inorganic chemistry and physical chemistry and I spent a considerable time during the undergraduate studies there, as a research and teaching assistant. I developed a special interest in electrolyte solutions, complex formation in water and the intriguing properties of water. When I was invited by the Head of Physical Chemistry, Erik Forslind, for a Ph D project studying by NMR the molecular aspects behind temperature anomalies of water, I accepted to join. Such temperature anomalies were very much discussed in the 1960s and had been inferred from several macroscopic observations (rheology etc.) by W Drost-Hansen and others [18]. Erik Forslind was a pioneer on NMR in Sweden and studied water and its electrolyte solutions by conventional ^1^H NMR [19, 20].

Discussing the Ph D project, it was argued that NMR relaxation rather than static NMR effects would be more sensitive for water structure effects and it was furthermore argued that ion NMR rather than conventional ^1^H NMR would be preferred in view of very large relaxation effects. At the department in Stockholm, there was a physicist Lars-Olov Andersson who performed ion NMR studies on solids and shared his knowledge on how to use the wide-line NMR experiment available.

The project showed to be a failure. Repeated attempts to do very detailed and accurate studies by several nuclei (^35^Cl, ^85^Rb, ^79,81^Br, ^23^Na, ^127^I) failed to indicate any special effects at the temperatures given in literature but gave throughout smooth and regular variations. A few years later, the reported temperature anomalies were found to be experimental artefacts [18].

A failed Ph D project is, of course, a big problem for a student as well as for the supervisor but fortunately there were several significant spin-offs. An important one was learning a new NMR technique, at that time only known by a few physicists and not applied to any chemical problems. In fact, I came to use it for a very large number of systems (surfactants, polyelectrolytes, proteins…) during the next decade.

**Fig. 2:**
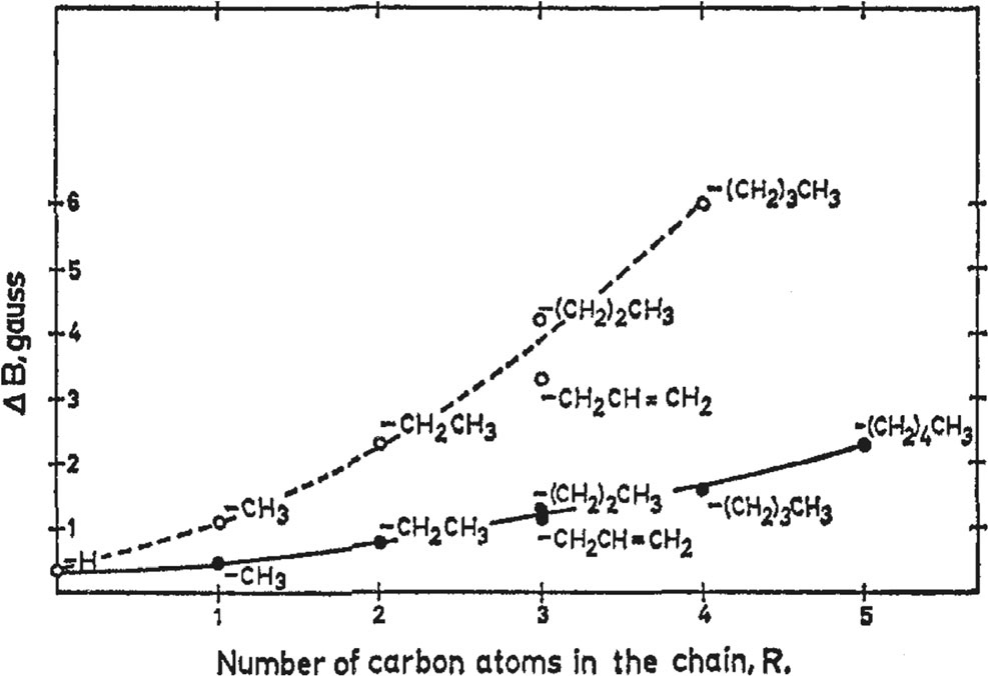
Quadrupole relaxation (here described by the NMR line width) of ^79^Br^−^ ions increases strongly in the presence of tetraalkylammonium ions (0.1 M for lower curve, 0.5 M for upper curve) demonstrating bromide ion association to hydrophobic cosolutes. Reprinted with permission from reference [21]. Copyright 2016 American Chemical Society

The NMR relaxation of most halide and alkali ions is dominated by quadrupolar interactions, i.e. interactions between the electric quadrupole moment of the nucleus and time-dependent electric field gradients arising from surrounding molecules and ions. In our work, the focus was on water structure effects so we were interested in ion–solvent effects. Ion–ion interactions, if large, could obscure this effect. Simple arguments lead to the conclusion that the oppositely charged ion should be as large as possible, as also indicated in literature on ion-pair formation. However, looking into Br relaxation in aqueous solutions of tetraalkylammonium bromides, a very striking opposite effect was found: Relaxation effects are orders of magnitude larger with organic than with inorganic counterions and increase strongly with the alkyl chain length (Fig. 2) [21].

These observations are significant since they directly demonstrate on a molecular level strong hydrophobic interactions in the binding of large anions. The observation was generalized to several organic counterions and it was also noted that there is a hydrophobic interaction also with uncharged and negatively charged species. Strikingly, the introduction of some polar character, like a hydroxyl group in a tetraalkylammonium ion, reduces strongly the association [22–24].

Control experiments for non-aqueous solvents showed that for this case the effect is the opposite: A large counterion gives small effects whereas a small one gives large effects.

This observation of the association between large anions and hydrophobic groups could certainly be inferred from other observations, like the Hofmeister series describing the effect of ions on the solubility of macromolecules, but it was here probably monitored directly for the first time. A few years later, we made use of this technique in studies of ion binding to different proteins [25]. From the binding sequence, it could be demonstrated that the Hofmeister series is related to direct association rather than indirect effects (“water structure”, etc.) [26, 27]. Rather extensive studies of ion binding to proteins etc. were performed and were reviewed in a monograph [28] and book chapters [29, 30].

Regarding the general mechanism of the association of ions due to hydrophobic interactions, a clear description has been given recently by Uri Sivan [31]. He describes for example the association of cations and anions to silica surfaces at different degrees of protonation; the analysis emphasizes the hydrophobic character of silica as is, of course, well-known from studies of surfactant adsorption (see below).

Ion binding sequences as described by the Hofmeister series are showing up in many colloidal systems and we have also encountered them in connection with clouding of polymer solutions, polymer adsorption and most recently cellulose dissolution. Some aspects are covered in a recent review [32].

## Ion binding in surfactant systems

Another spin-off of the initial Ph D work was a contact with Professor Per Ekwall. Ekwall pioneered surfactant studies at Åbo Akademi in Finland [33–37], and on retirement at the age of 67, he started the Laboratory of Surface Chemistry in Stockholm and initiated a large research program mainly dealing with ionic surfactants. When during my first year as Ph D student, he attended my seminar where I described the new quadrupole relaxation method for studying ion binding, he became very enthusiastic. We started immediately a collaboration on sodium ion binding in different isotropic and liquid crystalline surfactant phases [38, 39].

On transferring from Stockholm to Lund Institute of Technology (now part of Lund University), where Sture Forsén was new professor and built up a new excellent research environment, these studies were broadened together with two undergraduate students, Göran Lindblom and Håkan Wennerström. Largely due to the experimental efforts of Göran Lindblom and theoretical work by Håkan Wennerström, a general picture of electrostatic interactions in surfactant systems emerged. Håkan’s contributions included the realization that puzzling line-shapes in ^35^Cl NMR are the result of second-order quadrupole splittings [40]; the quadrupole splitting approach gave us a new powerful way of probing ion binding in liquid crystals [41]. Håkan also realized that the relaxation in colloidal systems must take into account motions on widely different time-scales and developed the “two-step model” [42]. Furthermore, the NMR studies stimulated theoretical work on electrostatic interactions. Håkan could rationalize an ion binding virtually independent of surfactant concentration in theoretical calculations based on the Poisson-Boltzmann equation [43].

## NMR techniques for surfactant and other colloidal systems

When we started to use NMR on surfactant systems, literature was limited to a small number of studies mainly based on ^1^H NMR chemical shifts, which are insensitive to the colloidal aspects. After our rather successful start with quadrupole relaxation of ions we soon added a large number of other approaches to our arsenal. These included:

- ion quadrupole splittings (mentioned above), which give direct information on the degree of ion binding, but can also report on intricate geometrical effects at the surfactant aggregate surface;
- ^13^C NMR chemical shifts for studies of alkyl chain conformation in micelles and on micelle aggregation numbers [44, 45]. It was for example found that the alkyl chains are only slightly more trans than in the unimeric solution and than in liquid alkanes;
- ^13^C NMR relaxation gives information on motions on different time scales. Important findings are about picosecond time-scale motions in micelles similar to that in liquid hydrocarbons as well as similar order parameters in micelles as in liquid crystals [46];
- Counterion chemical shift anisotropy. An important complement to quadrupole splitting and relaxation for those ions that do not have quadrupole moments [47];
- Self-diffusion coefficients, to be dealt with in a separate section;
- ^2^H NMR on deuterated water and deuterated surfactant for studies of hydration and chain conformation. Furthermore, it is powerful for phase diagram work;
- ^17^O NMR for hydration;
- ^19^F NMR relaxation and water penetration in surfactant micelles [48].

The NMR studies of the 1970s gave much of the basis for our attempts to clarify surfactant micellization in two reviews [49, 50]. Work on these reviews indicated for us existing gaps of understanding giving further research topics.

## Self-diffusion

During my early time as a Ph D student, Erik Forslind arranged for me a stay at the Technical University of Karlsruhe in the laboratory of Gerhard Hertz. Hertz was as Forslind a pioneer in the studies of electrolyte solutions by NMR and had in Münster made detailed studies by ^1^H and ^2^H relaxation [51–53]. In moving to Karlsruhe, he started also work on self-diffusion by the spin-echo technique. This technique was new and had not been used previously for studies of hydration and ionic interactions. With my experience of tetraalkylammonium halides, it was natural that I embarked on a study of these systems giving insight into hydration effects [54].

During my very useful stay in Karlsruhe in 1967, I could not imagine that NMR self-diffusion was a technique that would follow me into this decade. In fact, I have now published work using this approach in six decades.

At the time, such studies could not be performed in Sweden but soon this situation would change and then several problems in surfactant science were attacked, such as micellization, micelle hydration, microstructure of cubic phases and microemulsions, ion binding, etc. This is described below.

During my work on NMR self-diffusion in Karlsruhe it became clear that monitoring the translational motion of molecules or ions would be particularly informative for colloidal systems, like the surfactant systems we were investigating by ion NMR. However, the spin-echo NMR technique was then limited to simple systems and ions like sodium and chloride cannot be studied (because of relaxation effects). At a conference, I came in contact with a group at the University of Montpellier who studied self-diffusion of simple electrolyte solutions by a technique based on radioactive labelling. During a stay in Montpellier, I learnt the technique and studied (with Nicole Kamenka and others) the self-diffusion of water, surfactant ions and counterions in some micellar systems. In this way micelle formation could be sensitively monitored and information on hydration and counterion binding obtained using radioactively labelled compounds [55, 56].

The self-diffusion technique based on radioactive labelling is quite laborious and limited since it requires the use of radioactively labelled chemicals. Very important for the continuation of this work was the development of a new NMR technique, that could be used for complex systems without the need for any labelling. This is described below.

## Cubic, sponge and microemulsion phases

Per Ekwall was very pleased with our NMR work on surfactant systems and helped to publicize it, resulting for example in opportunities for presentations at important meetings. When he came in contact with G. W. Grey and P. A. Winsor about a new comprehensive treatise on liquid crystals, he arranged for me an invitation to write a chapter on “NMR on amphiphilic liquid crystals”; to this task, I invited a Ph D student colleague, Åke Johansson, to join [57]. The thorough reading of the (then quite limited) literature indicated several points of confusion, some leading directly to new research, others planting seeds for future. One such related to the structure of common surfactant cubic phases, where both V. Luzzati and Winsor had made important work but came to entirely different conclusions. Winsor saw a structure built up of spherical micelles as the most plausible one [58], whereas Luzzati advocated a mixture structure with connected network and discrete aggregates [59–62].

It was easy to realize that if we could measure surfactant self-diffusion, we could make a distinction. Thus surfactant diffusion with discrete particles would be slow whereas if surfactant aggregates extended over macroscopic distances, fast self-diffusion would be expected. During my stay in Montpellier as a postdoc, I planned such work but it proved that with the open-ended capillary tube technique, the very high viscosity of cubic phases made the study impossible. A couple of years later, we had received a spin-echo NMR spectrometer in Lund and one of the first things to do was to come back to this problem. The interest in cubic phases was still not very important and only a few phases were known; thus it was typically quite difficult to identify and separate cubic phases from other phases due to very high viscosity, optical isotropy and small density differences between phases. Krister Fontell (then at the Institute for Surface Chemistry in Stockholm) with his careful phase diagram work [63, 64] was one of the most prominent researchers in the field and he was generous to share his knowledge, this becoming a strong driving force in our research when he later moved to Lund University. From Krister, I learnt about the system of dodecyltrimethylammonium chloride-water, which has two cubic phases [65]. This appeared ideal for probing microstructure and in one afternoon’s experiment, postdoc Thomas Bull could show that the surfactant self-diffusion is completely different in the two phases [66]. Strikingly, the phase with lower surfactant concentration shows very slow diffusion, while the more concentrated phase has orders of magnitude faster diffusion. It was straightforward to identify one phase having discrete globular micelles and the other having three-dimensionally connected surfactant aggregates, i.e. being “bicontinuous”.

Our work on cubic phases brought us later in contact with Kåre Larsson, head of the Food Technology Division at Lund University. He was a pioneer on cubic liquid crystals with a focus on lipid systems rather than surfactants and also became later the pioneer on Cubosomes [67–77]. In the early 1980s, when Sven Engström started some work on practical applications of cubic phases, a collaboration with Kåre Larsson was established and a spin-off, Fluidcrystal AB, later renamed to Camurus AB, was established with an entrepreneur Gunnar Sandberg. After a modest start, even if one pharmaceutical product came to the market early, the company under the leadership of former Ph D student Fredrik Tiberg has in recent years become very successful (camurus.com).

**Fig. 3:**
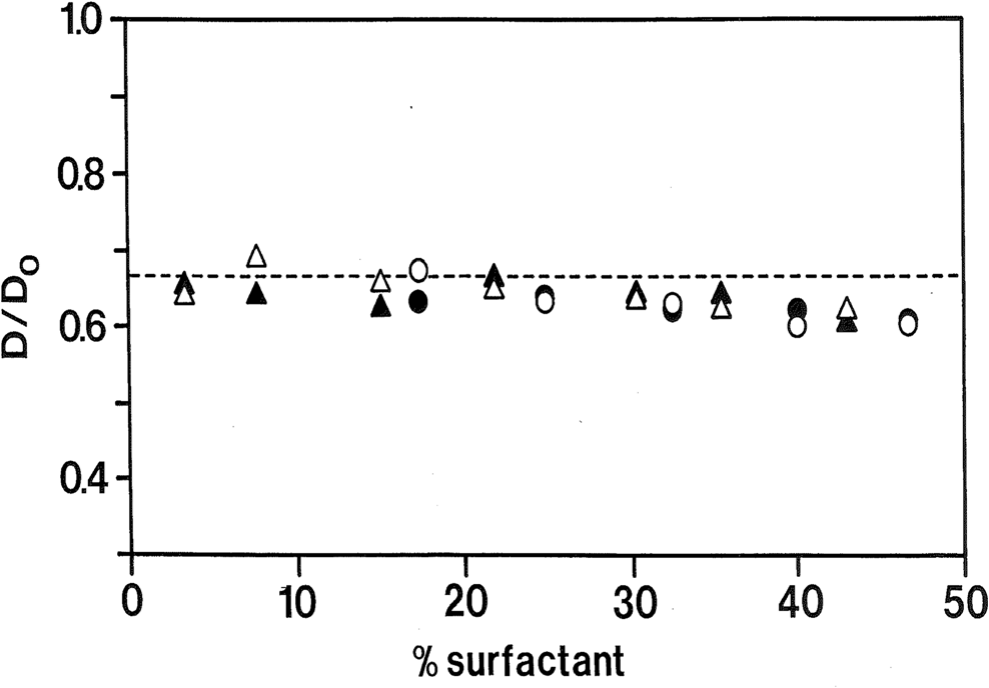
Bicontinuous surfactant phases were during a long period controversial. By molecular self-diffusion, they are easy to identify. Here are shown data (relative to the neat components) for water (*filled symbols*) and surfactant (*open symbols*) for sponge (L_3_) phases of two non-ionic surfactant systems. Both water and surfactant diffusion are very fast over wide ranges of concentration and close to 2/3 of that of the neat components, as predicted. Reprinted with permission from reference [79]. Copyright 2016 American Chemical Society

Regarding the simple surfactant systems, the L_3_ or “sponge” phase had been puzzling for a long time. In contrast to micellar surfactant systems, which show fast water and slow surfactant diffusion, the self-diffusion of both surfactant and water are here very fast, in fact close to 2/3 of the neat liquid compounds, over very wide concentration ranges (see Fig. 3). These observations provided direct evidence for a bicontinuous structure [78, 79].

Already much earlier, we had learnt about microemulsions, the concept called a misnomer by Ekwall, who saw these solutions as a natural consequence of surfactant phase behaviour, without a need for a special notion. Prominent work by Shinoda and Friberg (e.g. see reference [80]) and others [81, 82] showing large extensions of microemulsion regions indicated that the simple O/W or W/O pictures, dominating literature, could not apply. However, no techniques were available for structural characterization, the scattering techniques that had been so useful for micelles not being applicable at high concentrations of the “dispersed” medium. Here, the learning from diffusion in cubic phases became very handy and it could be predicted that it should be straightforward to distinguish between O/W, W/O and bicontinuous structures if we knew the oil and water self-diffusion coefficients. This can be done by radioactive labelling in the capillary tube technique or by using deuterated solvents and surfactant/cosurfactant in the conventional ^1^H spin-echo NMR technique. In our initial work, using a combination of radioactive measurements in Montpellier and NMR in Lund, we could directly demonstrate the occurrence of bicontinuous microemulsions in addition to the “classical” droplet structures [83]. However, with the need to label several components in complex mixtures this work became quite tedious and also had limited scope in terms of systems.

In the middle of this work, I received a paper for review from the rather unknown Swedish journal *Chemica Scripta* [84]. It was authored by Peter Stilbs, newly starting his research at Uppsala University, and it concerned a new approach of spin-echo NMR involving Fourier transformation, FT PGSE NMR. It allows the simultaneous determination of the diffusion coefficients of the components for complex mixed solutions. It immediately struck me that this was the remedy for our microemulsion experiments and I immediately called Peter to initiate collaboration [85]. Initially, the experiments were performed by Peter and his students in Uppsala, but in view of the demand, Peter kindly installed the technique also in Lund. An extensive collaboration on microemulsions as well as other surfactant systems started. (Peter has given a nice account of this [86]).

**Fig. 4:**
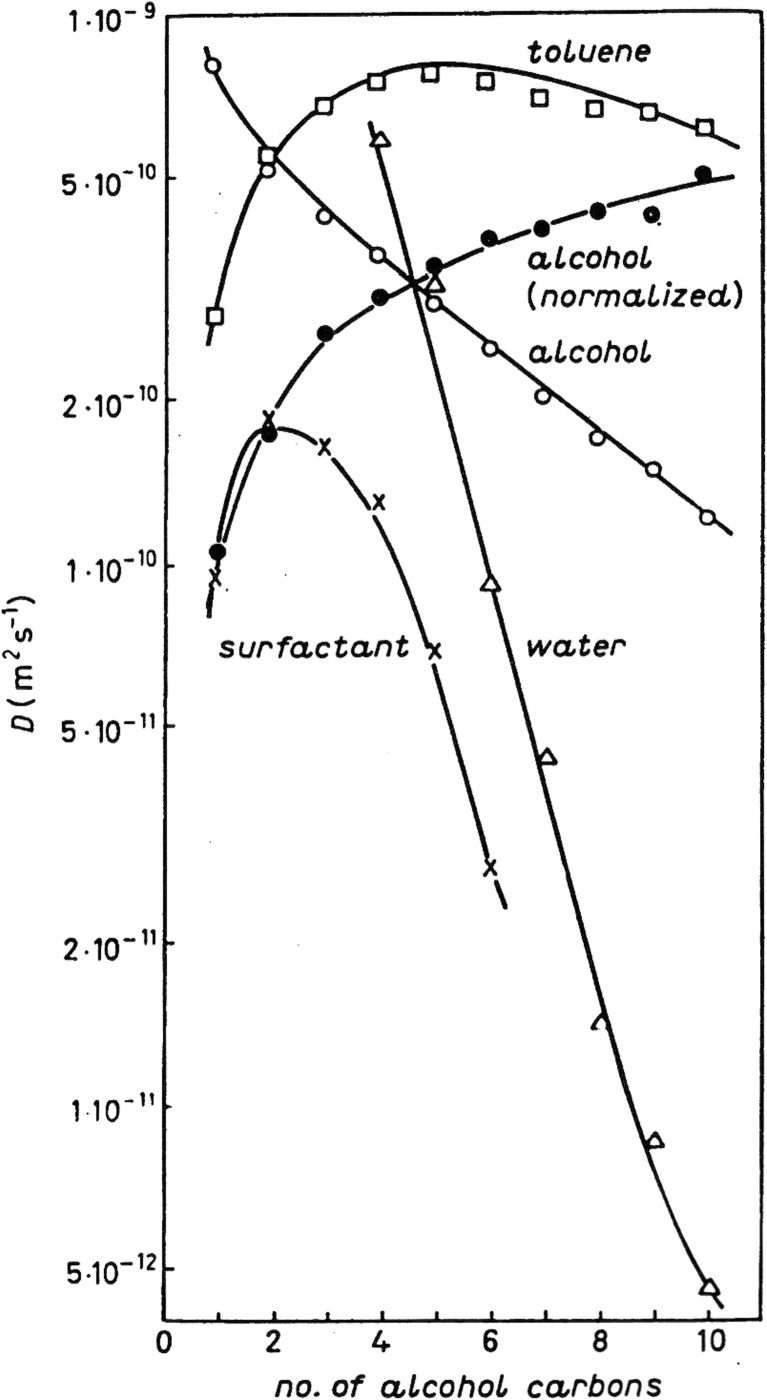
Self-diffusion coefficients in surfactant-alcohol-oil-water systems. For short-chain alcohols there is a bicontinuous structure (oil and water diffusion both fast) while for long alcohols water is confined into droplets. Reprinted from reference [87] with permission from Elsevier

Early work with Peter illustrated nicely the role of alcohol cosurfactant chain length in giving water-in-oil microemulsions (Fig. 4) [87].

**Fig. 5:**
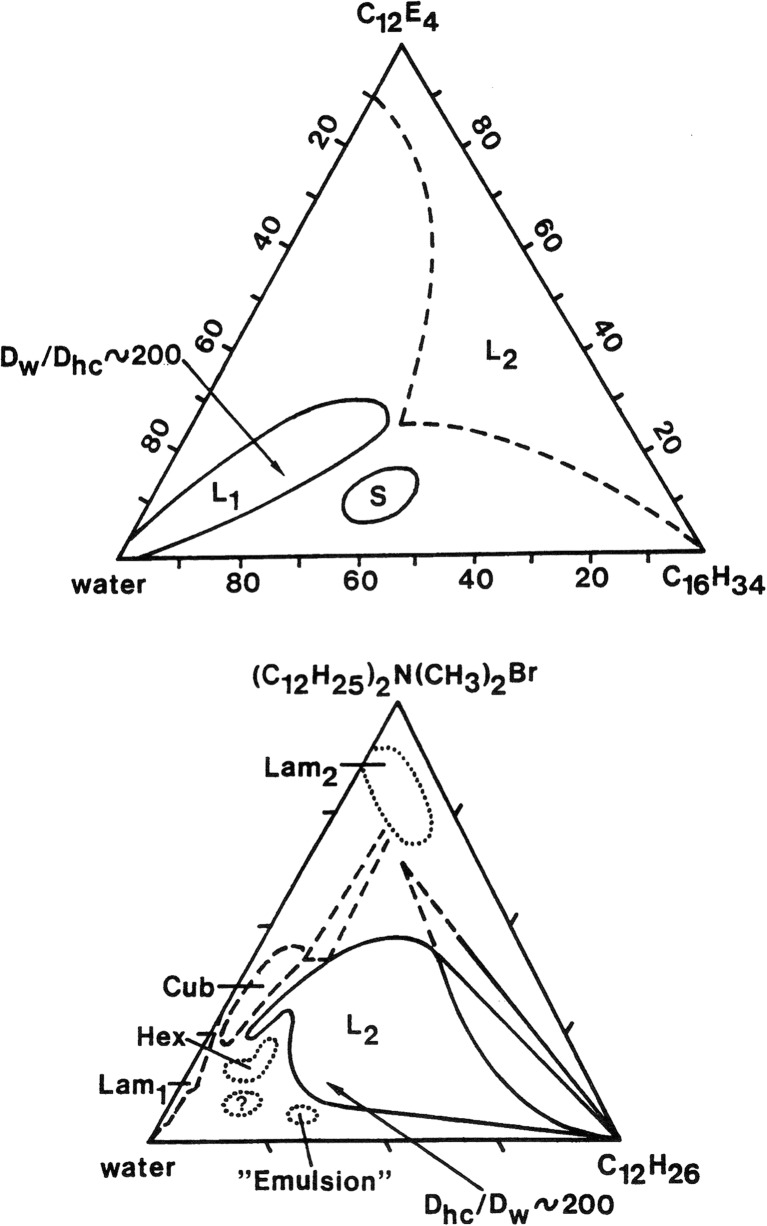
For the upper system we have O/W structure and for the lower W/O as shown by the hydrocarbon and water self-diffusion coefficients

Another important aspect of our work was the systems provided by leading researchers in the field, in particular Kozo Shinoda but also Dominique Langevin and others. A determination of the microstructure of a microemulsion is based on comparing the water and oil self-diffusion coefficients; often it is convenient to normalize by dividing with the values for the neat solvents. Our work is exemplified in the following figures. The power of the method can be described by the following figure (Fig. 5), showing orders of magnitude difference for similar compositions in two surfactant systems. Thus for the same composition, the ratio between oil and water self-diffusion coefficients may vary by almost 5 orders of magnitude between different surfactant systems.

**Fig. 6:**
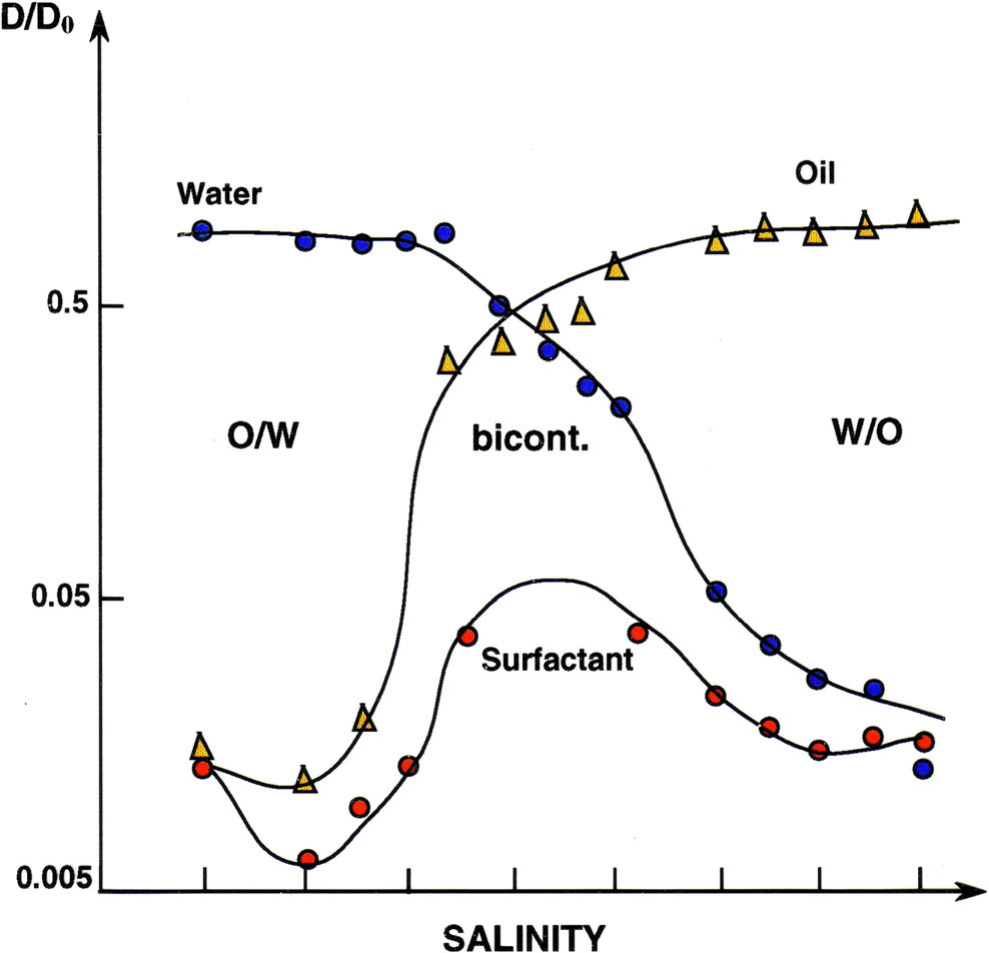
For ionic surfactant microemulsions, the microstructure changes from O/W to W/O via bicontinuous as salinity is increased [88, 89]. Reprinted with permission from reference [89]. Copyright 2016 American Chemical Society

**Fig. 7:**
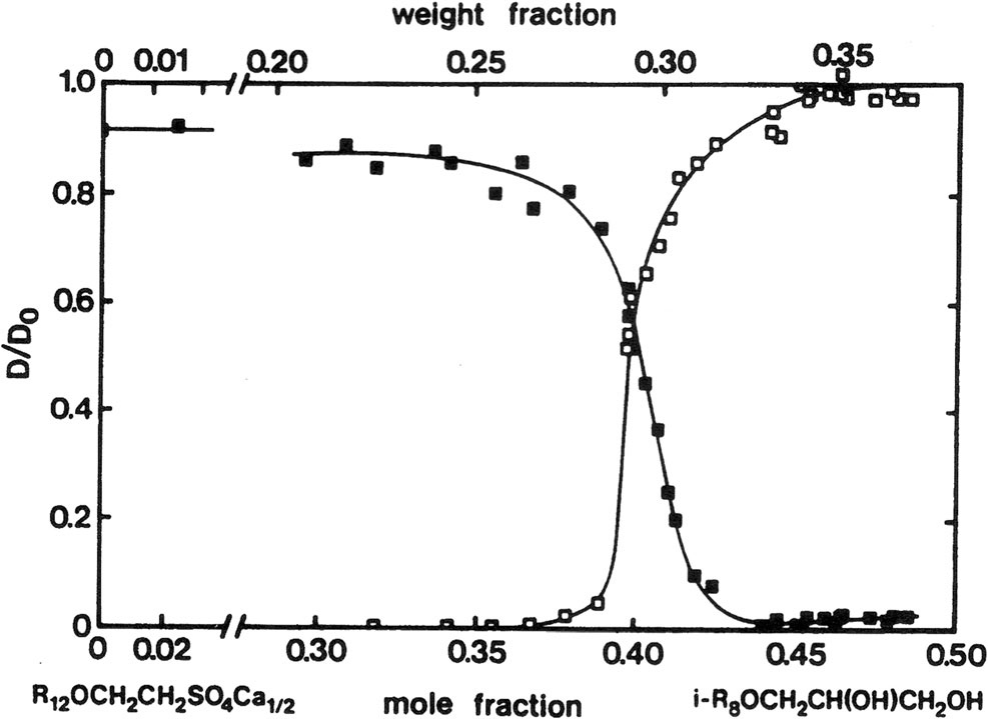
For mixed surfactant microemulsions the water (*filled symbols*) and oil (*open symbols*) self-diffusion coefficients change dramatically with surfactant composition. Reprinted with permission from reference [90]. Copyright 2016 American Chemical Society

**Fig. 8:**
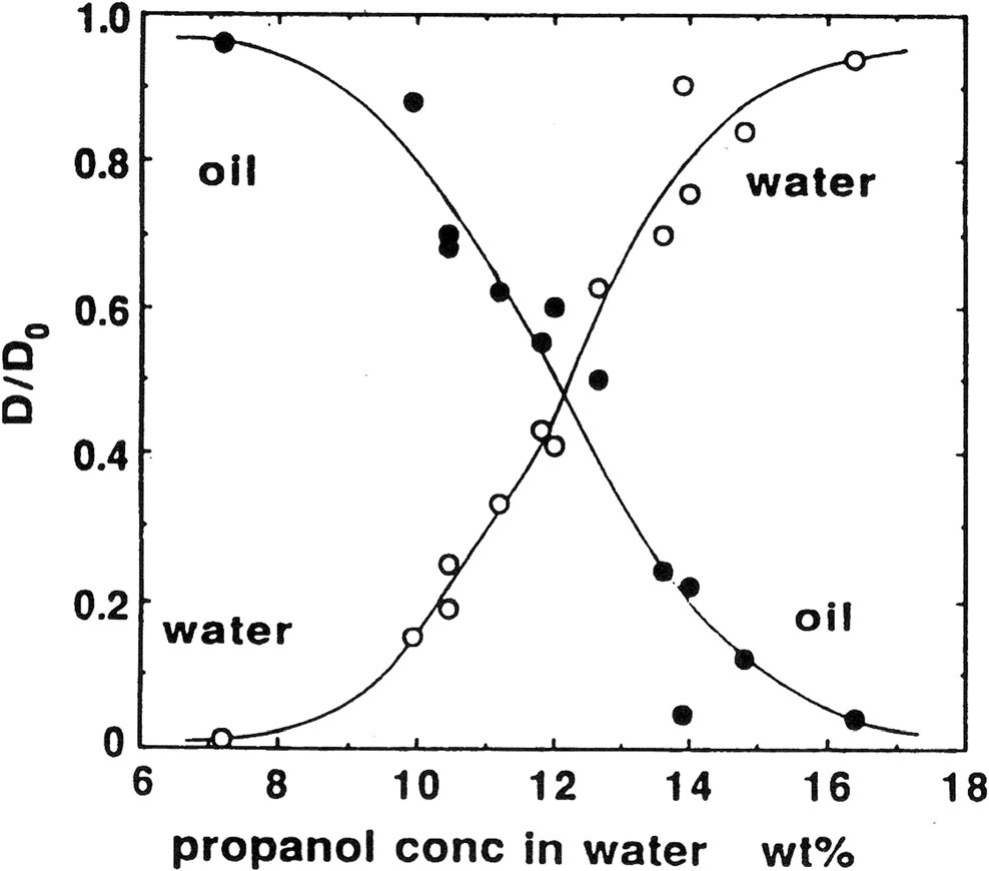
For these lecithin-based microemulsions, the propanol content determines microstructure. Reprinted with permission from reference [91]. Copyright 2016 American Chemical Society

The following figures (Figs. 6, 7, 8) show how microstructure depends sensitively on different parameters, on salinity for an ionic surfactant system, on surfactant composition for a mixed surfactant system and on cosolvent for lecithin microemulsions. For intermediate conditions, bicontinuous structures are encountered. Ideally, they are characterized by D/D_0_ equal to 2/3 for both water and oil.

The fact that a molecule confined to a micelle diffuses much more slowly than a molecule in the bulk solution had also other important applications, like for the determination of micellar compositions. This we had already used in the radioactive tracer experiments but now with the PGSE FT NMR technique studies of an extensive number of systems became feasible. Detailed information on hydration, counterion binding and solubilization could be obtained [92, 93].

## Phase diagrams by NMR

Phase diagrams are a fundamental part of surfactant science [94–97] not only because in any physico-chemical measurement we have to know if we are in a homogeneous phase or not but also because it gives a solid basis for understanding self-assembly; they are also the basis of applications. Surfactant phase diagrams are typically complex and difficult to explore since macroscopic phase separation is often hampered by the high viscosities and small differences in density between phases. A number of NMR parameters are very different for different phases and since NMR can be applied without the need of macroscopic phase separation (also turbid dispersions can be handled), there are good opportunities in surfactant phase science.

**Fig. 9:**
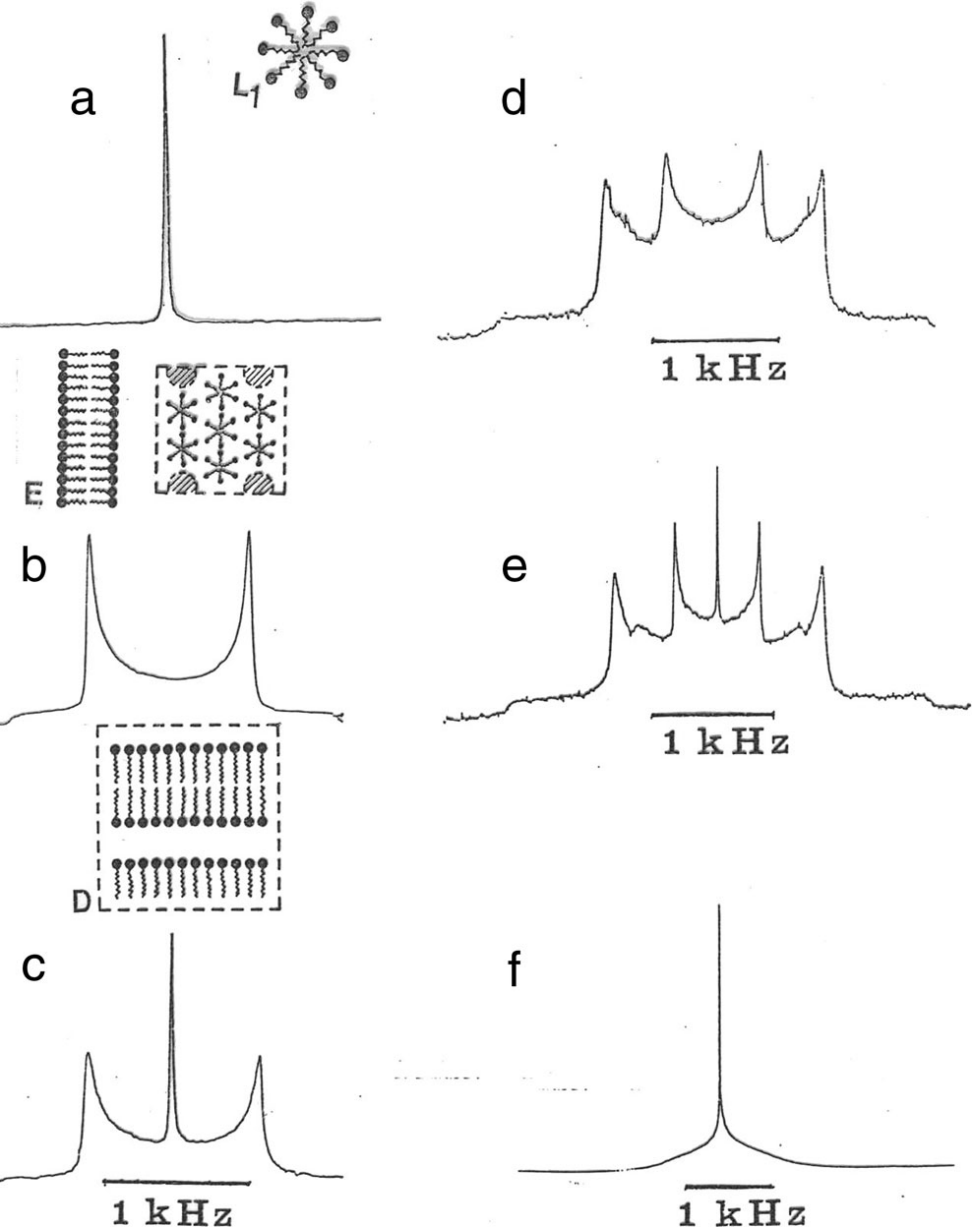
^2^H NMR spectra give quadrupole splittings for anisotropic phases, the lamellar phase giving larger splittings than the hexagonal one, and singlets for isotropic phases. For two- and three-phase samples there is a superposition of the spectra of the individual phases. **a** shows spectrum for an isotropic solution, **b** for a lamellar phase, **c** for coexisting lamellar + isotropic phases, **d** for coexisting lamellar + hexagonal phases, **e** for coexisting lamellar + hexagonal + isotropic phases and **f** for a fine dispersion of liquid crystalline phase. Reprinted with permission from reference [98]. Copyright 2016 American Chemical Society

Whereas several nuclei and NMR parameters can be useful, ^2^H NMR on samples enriched in deuterated water is the most versatile and has been widely applied, also for polymer systems [98]. Since a quadrupole splitting occurs for anisotropic environments but not for isotropic phases and since lamellar and hexagonal phases give different magnitudes of the splitting, it is as illustrated in Fig. 9, easy to map out one-, two- and three-phase regions, as well as to determine the identities of the phases.

The ^2^H quadrupole splitting technique has also been instrumental in mapping the complex phase behaviour of block copolymer systems, to demonstrate the coexistence of two lamellar phases, to map the counterion-dependent swelling of lamellar phases as well as to clarify intriguing three-dimensional phase diagrams of mixed cationic–anionic surfactant systems. Interestingly, the first demonstration of the usefulness was somewhat accidental. In investigations of different phases in Ekwall’s “holy system” sodium octanoate–decanol–water, we observed line-shapes for the “C phase” that were inconsistent with a single phase; a closer analysis revealed that it is a very stable dispersion of the lamellar phase in water [99].

**Fig. 10:**
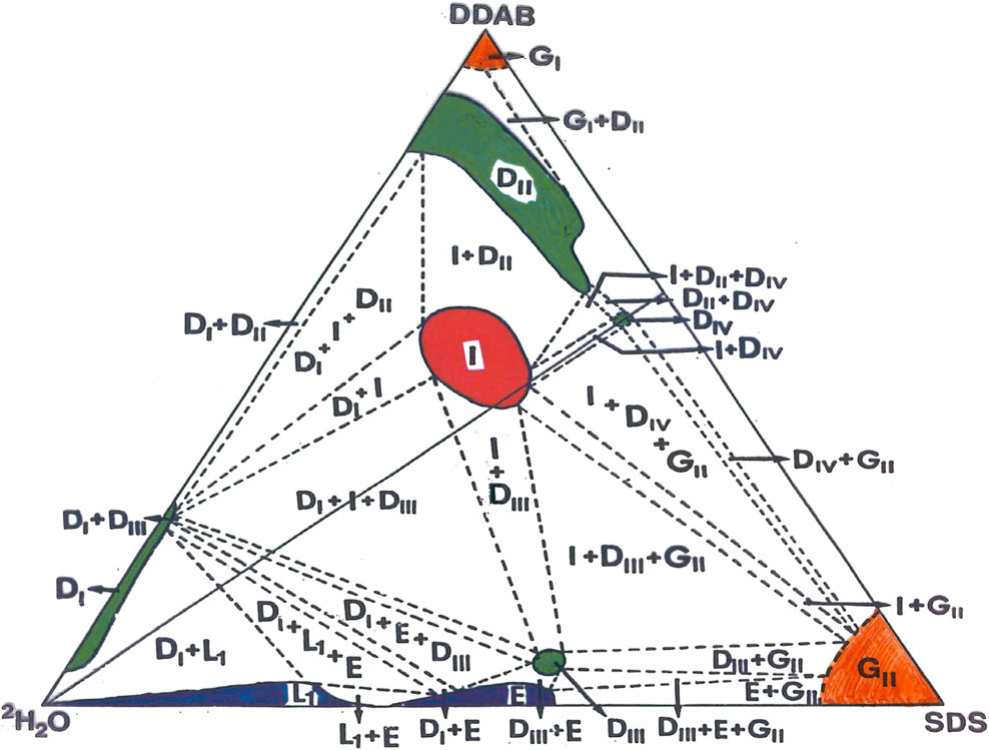
Mixed systems of cationic and anionic surfactants show a complex phase behaviour. Phase diagram determination has been much helped by ^2^H NMR. Reprinted with permission from reference [100]. Copyright 2016 American Chemical Society

One of the phase diagrams that have been determined on this basis is presented in Fig. 10. It concerns a mixture of a cationic and an anionic surfactant. Important features illustrated are that of thermodynamically stable vesicles, several lamellar phase regions as well as a very large number of multi-phase regions. It should be pointed out that a full representation of such a system would require a three-dimensional diagram.

Block copolymers in mixtures of two immiscible solvents have a rich and intriguing phase behaviour. The large number of phases as well as two- and three-phase regions could be conveniently located using ^2^H NMR [101, 102].

## Micelle structure and size

When we started to study surfactant systems, even basic aspects of the simplest micelles were unclear. There were controversies regarding the packing and dynamics of the alkyl chains, the degree of water penetration into micelles and the location and binding of counterions. Large efforts by several groups were directed to such problems in the 1970’s partly with quite a large focus on studying “probes”, which could “report” on parameters like “microviscosity” and “local polarity”. These probes would of course always perturb the system to a marked extent, and in several cases it was clear that this leads to highly misleading conclusions.

Here, NMR had major advantages as it requires no probes and measurements are done directly on the real systems. ^13^C NMR chemical shifts showed that the alkyl chain conformation in micelles is very much the same as in the non-associated molecules and in liquid hydrocarbons but quite different from that in solid hydrocarbons. ^13^C NMR relaxation, when properly interpreted using the Wennerström two-step model, showed that there are chain motions only slightly slower than in liquid hydrocarbons. The motions are slightly anisotropic giving an order parameter similar to that of the liquid crystals [46].

The question of surfactant hydration in micelles and “water penetration” into the interior of micelles was a long-term controversy. Probe methods were taken to infer a considerable amount of water deep into micelles. However, NMR self-diffusion measurements could quantify hydration and gave hydration numbers corresponding to hydration of the polar heads alone and the same could be inferred from ^17^O NMR relaxation and ^2^H NMR quadrupole splittings, the latter for the corresponding liquid crystalline phases [103, 104].

From intermolecular relaxation effects between the hydrophobic chains and water, the presence of water molecules along the chains could be quantified. It was found that water contact only occurs for the α-C but is negligible away from the head-group. The misleading information from probe techniques could be attributed to the fact that the probes used had an amphiphilic character leading to probes being preferentially located close to the aggregate surface.

Regarding micelle size, there were advocates for micelles always being spherical and for various non-spherical shapes and micelle–micelle aggregation. The phase diagram work had revealed that, depending on conditions, a large number of structures are possible, while the work on micellar solutions by Luzzati and others had clearly demonstrated that depending on conditions (concentration, alkyl chain length, salt, temperature), micelles can be small (spherical) or large (referred to as rod-like, thread-like, cylindrical) [105, 106].

Whereas the situation appeared clear for ionic surfactants, it was controversial for non-ionics. Micelle size, micelle aggregation, phase separation and critical fluctuations are important effects for any surfactant system. Different experimental quantities may be sensitive to one or more of these. Confusion arose since this was not always critically examined. In particular, scattering data were interpreted either in terms of micellar growth or critical fluctuations without properly considering both factors. Thus scattering techniques that were so important for ionic surfactants need much more careful analysis for non-ionic ones. While scattering was first interpreted in terms of massive growth, the tendency then went too far and scattering data were analysed without allowing for any growth.

**Fig. 11:**
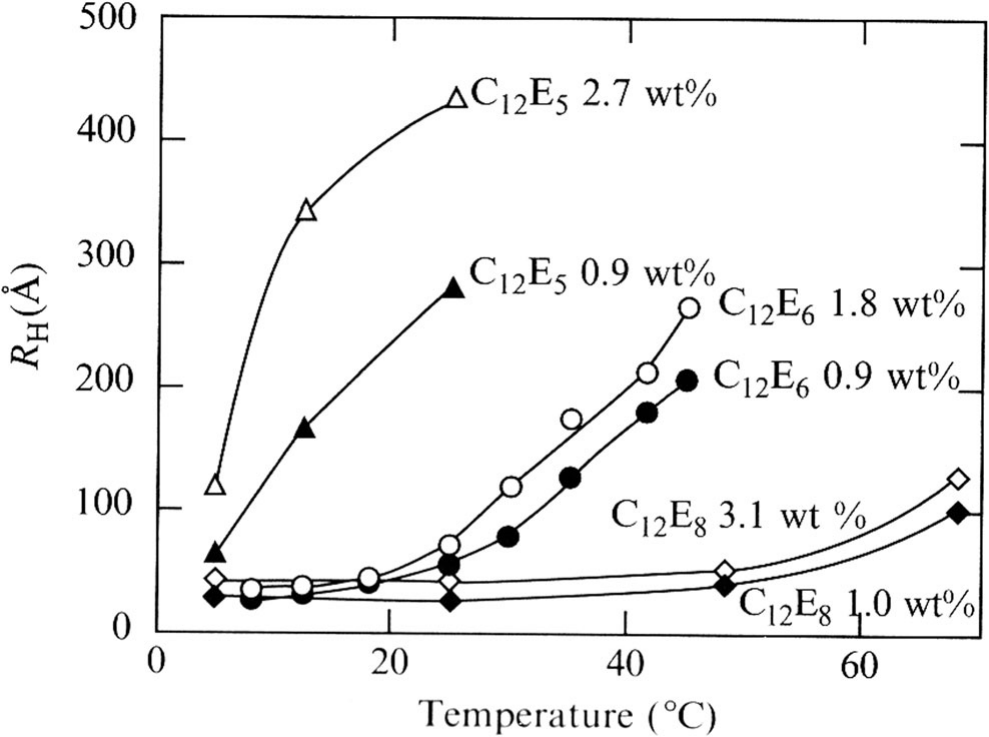
The micelle hydrodynamic radius (derived from surfactant self-diffusion) of non-ionic surfactants is strongly dependent on temperature and head-group [107, 108]. Reprinted with permission from reference [107]. Copyright 2016 American Chemical Society

At an early stage, it was realized that different NMR techniques could contribute to resolve the issue, namely NMR self-diffusion and NMR transverse relaxation. NMR effects are insensitive to critical fluctuations (and even to macroscopic phase separation) and relaxation also to intermicellar interactions; NMR relaxation is very much affected by major growth. The early NMR studies clearly demonstrated that there may be a major micellar growth with temperature but also that the effect depends very much on the head-group size; for a surfactant like C_12_E_5_, there is major growth, whereas growth is insignificant over a wide temperature range for C_12_E_8_ (Fig. 11).

It was also found that growth is not directly related to the distance from phase separation; e.g. the cloud point (CP) may be increased significantly by small amounts of ionic surfactant, while micelle size is not significantly affected. Phase separation, micelle aggregation and micelle growth depend on the same molecular interactions namely the worsening of EO–water interactions with increasing temperature. Because of the concomitant reduced hydration, the effective head-group area decreases (inducing micellar growth), intermicellar repulsion decreases (giving micelle association) and the water–surfactant miscibility decreases (giving phase separation).

As said above, the 1970s and early 1980s was a period of intense research on micelles and fundamentals of surfactant self-assembly world-wide. New experimental techniques and new theoretical models were proposed [109–114]. During this period, we organized in Lund in 1982 the first conference under the name Surfactants in Solution (under the chairmanship of Kash Mittal this biannual series of conferences is continuing, the latest one organized in Jinan, China, in 2016 and the next one planned for Oklahoma 2018). The three-volume proceedings of this conference “Surfactants in solution” [115] document well the existing controversies and new advances (for example [116–127]).

Discussions on micelle structure and dynamics continued during several years. Interestingly, a general outcome [128] was that early suggestions by Hartley [129, 130] were basically confirmed.

## Polymer–surfactant systems

**Fig. 12:**
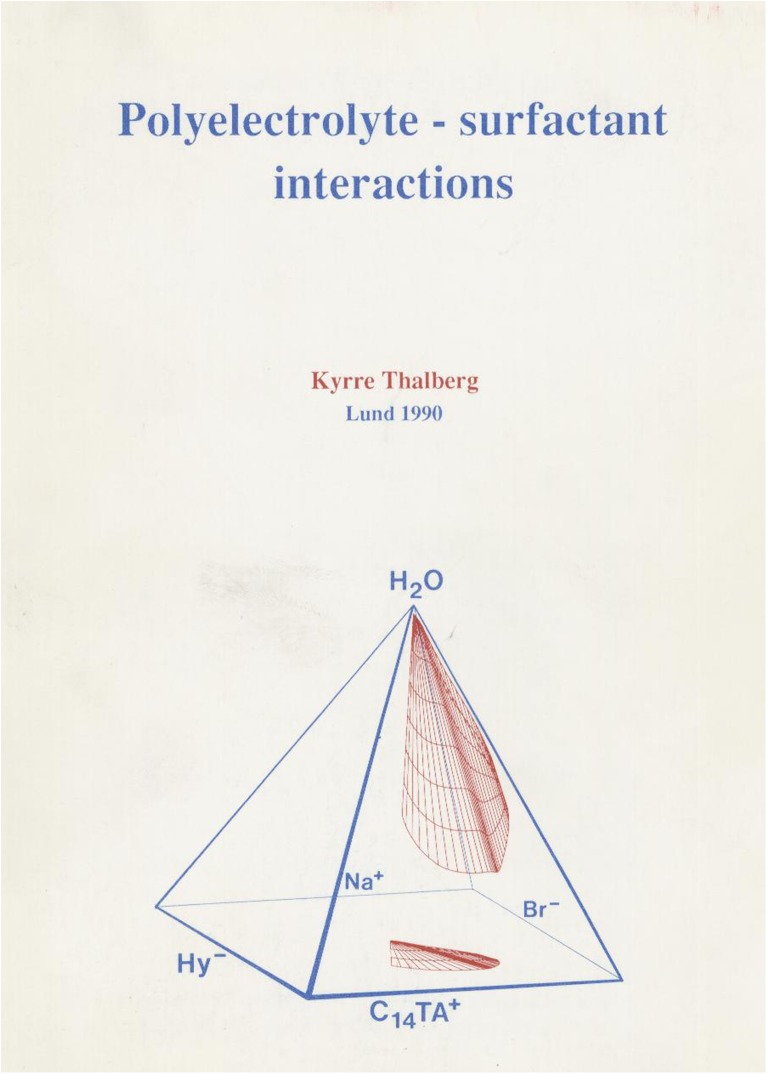
Kyrre Thalberg’s Ph D thesis involved original work on polyelectrolyte-surfactant systems, an area that has continued to be of importance in our research group in Lund

Our research in colloid science was initially very much focussed on NMR methodology and NMR studies of “simple” surfactant systems. When, in 1978, I became full professor and head of the Physical Chemistry 1 division at Lund University, a broadening of the scope and methodology was seen as important. Therefore, progressively we added new techniques (light scattering, SAXS, rheology, ellipsometry and other surface techniques, etc.) and new systems (polymer solutions and gels, surface-active polymers, polymer and surfactant adsorption, detergency, disperse systems etc.) to our research program. In several cases new research ventures came up due to industrial contacts and/or funding. A clear example is the field of polymer–surfactant interactions where contacts with AkzoNobel (previously Berol and BerolNobel) and Pharmacia in the early and mid-1980s clearly showed the significance of mixed polymer–surfactant systems in formulations; AkzoNobel was interested in non-ionic cellulose derivatives (water-based paints, etc.) and Pharmacia in hyaluronic acid (ophthalmic applications). With the Ph D students Anders Carlsson and Kyrre Thalberg, we embarked on original phase diagram and structural studies. Anders Carlsson clarified the clouding and phase behaviour of mixtures of non-ionic polymers and ionic surfactants [131, 132] and discovered new thermal gels [133] (that were exploited in a new company, Kabi Invent AB) whereas Kyrre Thalberg thoroughly characterized the intricate phase behaviour of polyelectrolyte-oppositely charged surfactant systems (Fig. 12) [134, 135]. An especially strong relation was established with AkzoNobel in Stenungsund in Sweden, which financed a number of Ph D projects; I was the chairman of their research council for many years.

On the basis of this modest start a huge research activity on polymer–surfactant systems developed with several colleagues being instrumental (Lennart Piculell, Ulf Olsson, Tommy Nylander….). Both bulk and interfacial aspects were covered and both fundamental and applied aspects. The research benefitted strongly from many industrial contacts, like AkzoNobel, Procter&Gamble, BASF, Kao, GlaxoSmithKline, Cognis, etc.

Important aspects of the polyelectrolyte-ionic surfactant work were the strong associative phase separation, the influence of charge density and electrolyte, and the need to describe the phase behaviour in three dimensions.

When we started our research on mixed polymer–surfactant systems, there had been important contributions for example by Goddard [136], Cabane [137] and a few others [138] but work outside a few industrial research laboratories was quite limited.

**Fig. 13:**
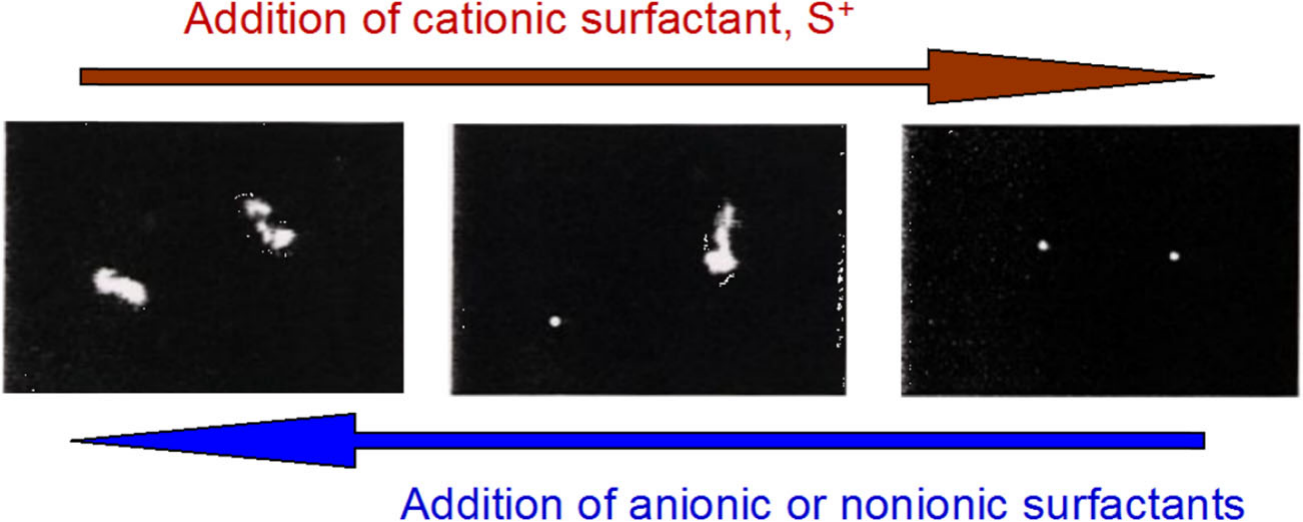
Addition of cationic surfactant induces compaction of extended DNA into globules as observed by fluorescence microscopy. Compaction is reversed by anionic, and less strongly by non-ionic, surfactants because of formation of mixed surfactant aggregates. Adapted from reference [139] with permission from Wiley

**Fig. 14:**
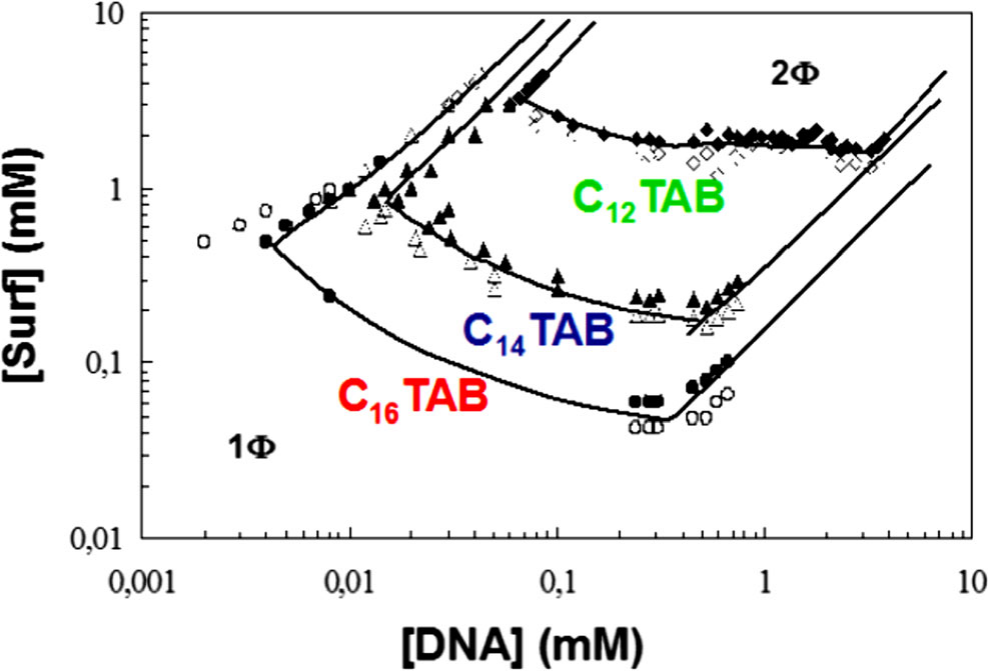
Schematic phase maps of mixtures of double stranded DNA and cationic surfactants. There is an associative phase separation from low surfactant concentrations. Reprinted with permission from reference [140]. Copyright 2016 American Chemical Society

The work on polyelectrolyte-surfactant systems motivated us to start work on DNA. These studies got much more strength when Sergey Mel’nikov from Kenichi Yoshikawa’s laboratory in Kyoto came to us as a postdoc. It became a good opportunity to combine Sergey’s experience on DNA compaction with ours on polyelectrolyte-surfactant interactions in general, including phase diagrams. We had previously studied the interactions between surfactants and oppositely charged polyelectrolytes with strong or weak hydrophobic character; the significant hydrophobic properties of DNA, as illustrated by its self-assembly (double-helix, etc.), showed to be very important for the interaction with surfactants. At the time, we had established close contacts with Maria Miguel’s group at the University of Coimbra and with Eduardo Marques, making a thesis on mixed cationic-anionic surfactant systems (cf. Fig. 10), as the first there was an intense student exchange Coimbra–Lund starting (and still going on). Our second Coimbra Ph D student, Rita Dias, started in parallel work on DNA compaction by cationic surfactants using fluorescence microscopy (Fig. 13) [139] and phase diagram work (Fig. 14) [140]. The latter revealed, as expected, a strong associative phase separation. However, there were also interesting novel observations. Thus single-stranded DNA showed to associate more strongly to cationic surfactants than the double-helix form; this shows the significance of the exposed bases and the polymer flexibility for the association. A puzzle was that adding excess surfactant did not lead to redissolution as expected for a polymer with hydrophobic properties. It was only much later with the help of modified mixing schemes that this matter could be explained in terms of kinetic trapping [141].

**Fig. 15:**
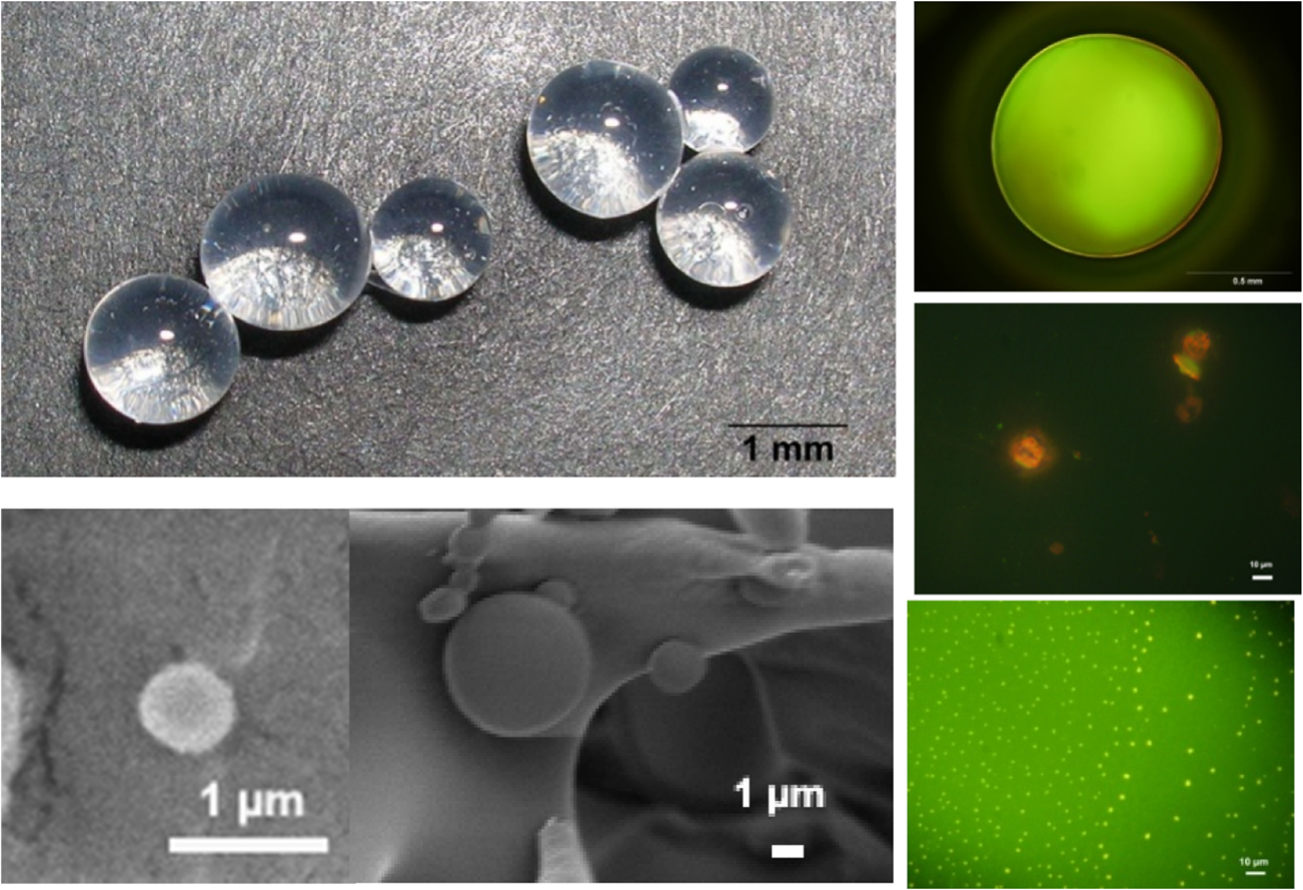
DNA gel particles prepared on the basis of associative phase separation. By the courtesy of Carmen Morán

The DNA work, mainly in Coimbra–Lund collaborations but also involving other partners, came to involve many aspects like modelling, interfacial aspects, covalent and physical gels etc. [142]. As an example, gel particles of widely different sizes can be prepared on the basis of the strong association between DNA and cationic compounds (surfactants, proteins, polymers) [143, 144]. These particles are illustrated in Fig. 15.

## Interfacial and adsorption aspects of polymers and surfactants

Work in our group on interfacial aspects started with a Ph D thesis by Kjell Bäckström on cleaning of hard surfaces, supervised by Sven Engström and in collaboration with the Division of Food Technology (Kåre Larsson, Tommy Nylander and Thomas Arnebrant), which had developed a very useful in situ ellipsometry methodology. This Ph D thesis demonstrated a new way of monitoring detergency and demonstrated how fat removal is related to surfactant packing at interfaces [145, 146]. Here and in a follow-up study by Martin Malmsten, it was illustrated from work on inter alia surfactant mixtures that a critical packing parameter around 1 is optimal for removal [147]. In some later industrially sponsored work, we have returned to such studies.

The ellipsometry technique stimulated very much our plans for broad studies of polymer and surfactant adsorption. Methodological developments by Bengt Jönsson, Mikael Landgren and Tommy Nylander laid the ground for the Ph D theses of Martin Malmsten (see, for example, references [148, 149]) and Fredrik Tiberg (see below), focussing on polymer and surfactant adsorption, respectively. Much of the later work concerned mixed polymer–surfactant systems, with a thesis by Fredrik Joabsson (for example, see reference [150]) on mixed systems of non-ionic cellulose derivatives and ionic surfactants and much work by Tommy Nylander and students and coworkers on polyelectrolyte-surfactant systems (Yulia Samoshina, Eiji Terada, Marité Cárdenas etc.).

**Fig. 16:**
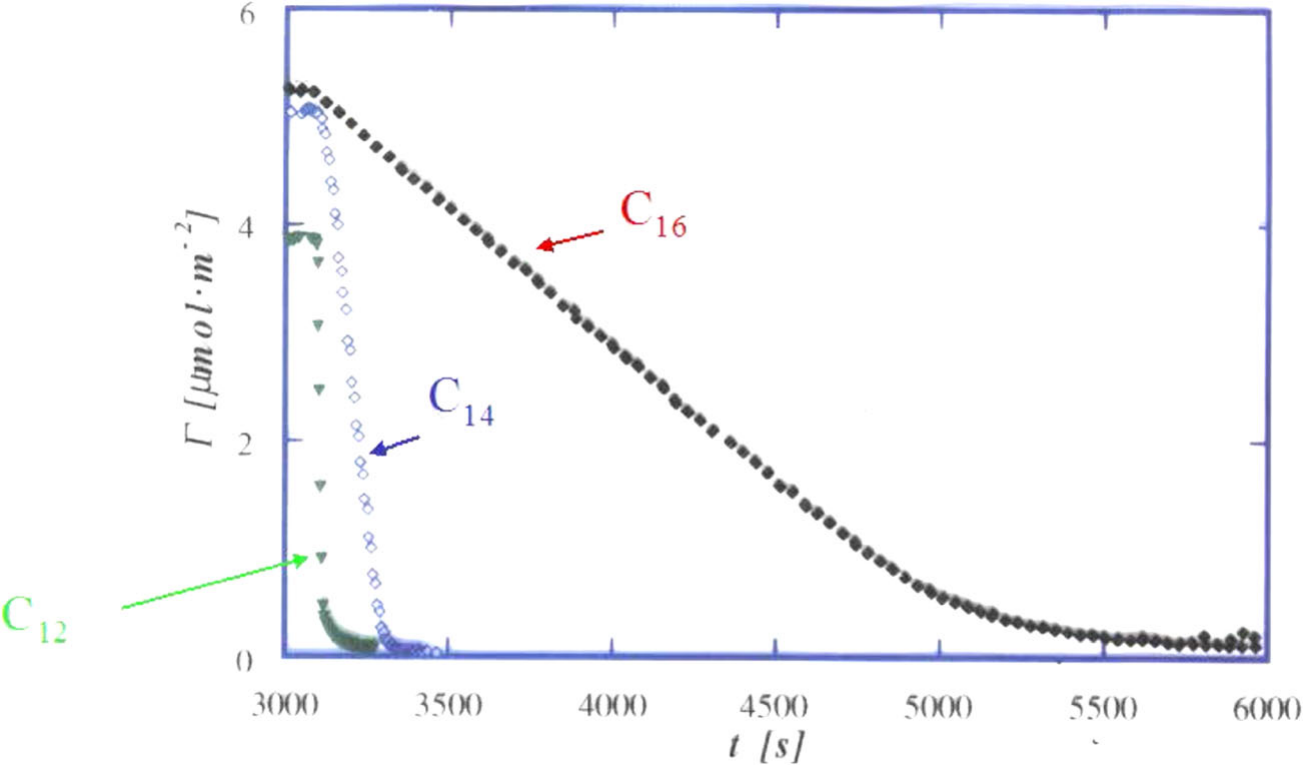
For a long-chain surfactant, desorption is quite slow which is due to the strong interfacial self-assembly. Reprinted with permission from reference [152]. Copyright 2016 American Chemical Society

Most of the ellipsometry work is performed for silica surfaces; these have distinct both hydrophobic and hydrophilic properties and thus are amphiphilic [31]. This leads to strong adsorption of surfactants as well as of polymers, if these have some hydrophobic character. For surfactants, adsorption is strongly cooperative and involves surfactant self-assembly (in contrast to hydrophobic surfaces where surfactant adsorption is non-cooperative and does not involve self-assembly). This is clearly illustrated from the effect of alkyl chain length on both equilibrium adsorption and the rate of desorption. The surface CMC varies with the alkyl chain in very much the same way as the bulk CMC [151]. Surfactant desorption is due to the transport of individual surfactant molecules from the surface; it is quite slow for a surfactant with a low (surface) CMC (Fig. 16). Interfacial aggregation is confirmed since the desorption rates follow the adsorption isotherms [152].

That adsorption of non-ionic polymers is typically determined by solvency effects was clearly demonstrated in Martin Malmsten’s work [153]. So, for example, the relation between polymer adsorption and anions in the Hofmeister series showed a strong correlation between adsorbed amount and the effect of ions on polymer solubility (cloud point); whereas iodides, for example, decrease adsorption, chlorides enhance it.

**Fig. 17:**
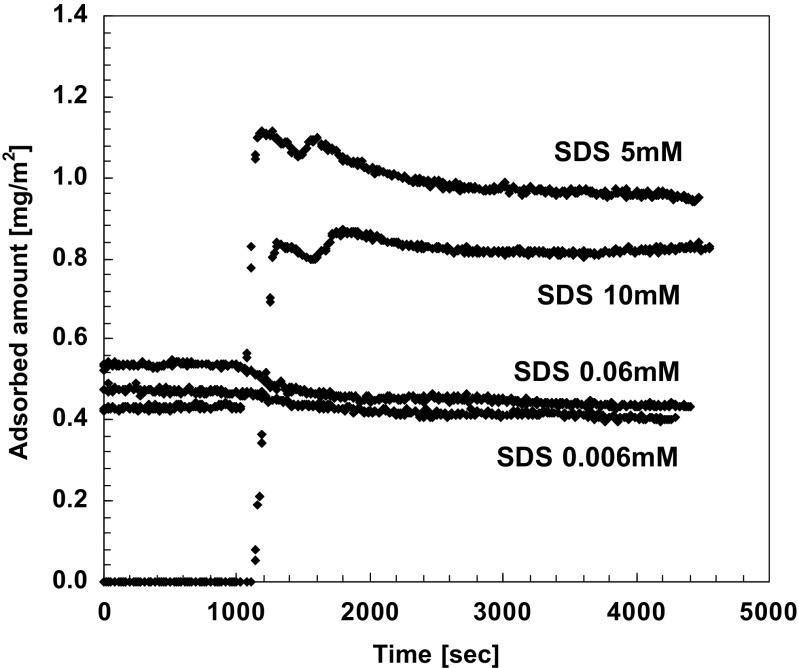
Deposition of cationic polymers from mixed anionic surfactant-cationic polymer systems monitored in situ and described on the basis of phase behaviour providing a basis for applications in hair-care. It is shown that dilutions of solutions of anionic surfactant (sodium dodecyl sulphate, SDS, in charge excess) + cationic polymer (cationic hydroxyethyl cellulose) may lead to deposition. Rinsing starts at time 1,000 s. Reprinted with permission from reference [154]. Copyright 2016 American Chemical Society

Solvency effects are particularly striking for mixed polyelectrolyte-ionic surfactant systems and also have important applications. As described above, there is a strong associative phase separation in such systems; if the polymer has some hydrophobic character, excess surfactant leads to redissolution. Strikingly, if such redissolved systems are diluted, adsorption on a surface occurs because the system enters the two-phase diagram. (Illustrated in Fig. 17.) This effect can be used in applications (hair-care, etc.) for polymer deposition.

## Scandinavian and European networks

Above, I have emphasized the starts of different research areas in our division at Lund University where I have been involved. Initiatives have been taken together with several colleagues and coworkers, who often have followed up with deep-going successful work. In the start of my career, Scandinavian contacts were dominating and in particular I benefited from contacts with the pioneering research at Åko Akademi (started by Per Ekwall and continued by Ingvar Danielsson, Per Stenius and Jarl Rosenholm) (cf. reference [155]) During long periods, there were very fruitful contacts with the Institute for Surface Chemistry in Stockholm, especially under the directions of Per Stenius and Krister Holmberg. Scandinavian contacts dominated at an early stage; so for example, there were important Scandinavian meetings initiated by Per Ekwall already in the early 1960s. International contacts were also important from an early stage, most important being those with Japan, France, Israel and Italy, where in particular Maura Monduzzi spent several periods in Lund (cf. see reference [156]).

European research activities were early on rather scattered, partly due to lack of meeting places. During the beginning of the 1980s the situation changed and notably Italian and French colleagues arranged important conferences and we contributed with the Surfactants in Solution meeting in Lund in 1982 (see above). With increasing European research activities, the need for a regular forum was brought up when Heinz Hoffmann (Bayreuth), Vittorio Degiorgio (Milano) and myself met on a conference in Manchester. Based on our discussion and with support of Pierre Bothorel (Bordeaux) and Mario Corti (Milano), the European Colloid and Interface Society (ECIS) was founded. Mario and Vittorio organized the first meeting in Varenna in 1987. (Other important early driving forces in ECIS include Gerd Olofsson, Jarl Rosenholm and Peter Schurtenberger.) ECIS has developed into a very strong association and with very well-attended annual conferences; this year the 30th ECIS conference is held in Rome.
